# Tongue Root Cyst as a Manifestation of the Variant m.3243A>G

**DOI:** 10.7759/cureus.19060

**Published:** 2021-10-26

**Authors:** Josef Finsterer

**Affiliations:** 1 Neurology, Krankenanstalt Rudolfstiftung, Vienna, AUT

**Keywords:** cyst formation, oxidative phosphorylation, mtdna, melas, m.3243a>g

## Abstract

There are indications that the frequency of cyst formation is increased in mitochondrial disorders (MIDs). Cysts can be found in various organs of MID patients but tongue root cysts have not been reported as a manifestation of a MID.

In a 56-year-old male with mitochondrial encephalopathy, lactic acidosis, and stroke-like episodes (MELAS) manifesting with recurrent stroke-like episodes, seizures, cognitive impairment, ataxia, psychiatric abnormalities, bilateral visual impairment, bilateral hypoacusis, pre-diabetes, hyperlipidemia, myopathy, and lactic acidosis, an asymptomatic, pre-epiglottic cyst with 1 cm in diameter and protein-rich content in the right tongue root was accidentally detected on ultrasound and confirmed by MRI at the age of 50 years. The patient did not complain about dysarthria or dysphagia but had a mild cerebellar speech. The cyst was confirmed on an MRI six years later, without having changed in diameter, extension, or symptomatology. Due to the atypical location, a branchiogenic cyst was excluded. In conclusion, tongue root cysts may be a manifestation of the m.3243A>G variant.

## Introduction

There are indications that the prevalence of cyst formations is increased in patients with a mitochondrial disorder (MID) [1]. Cysts can be found in various organs of MID patients, particularly in the brain, kidneys, liver, pancreas, and ovaries [2-4]. Whether cysts also occur in the striated or smooth muscles is so far unknown. The cause of the apparently increased frequency of cyst formation in MIDs is unknown but several speculations have been raised to explain the phenomenon [4]. Cysts in the tongue have not been reported as a manifestation of a MID so far. Here, we present the first patient with a MID and a cyst in the tongue.

## Case presentation

**Figure 1:**
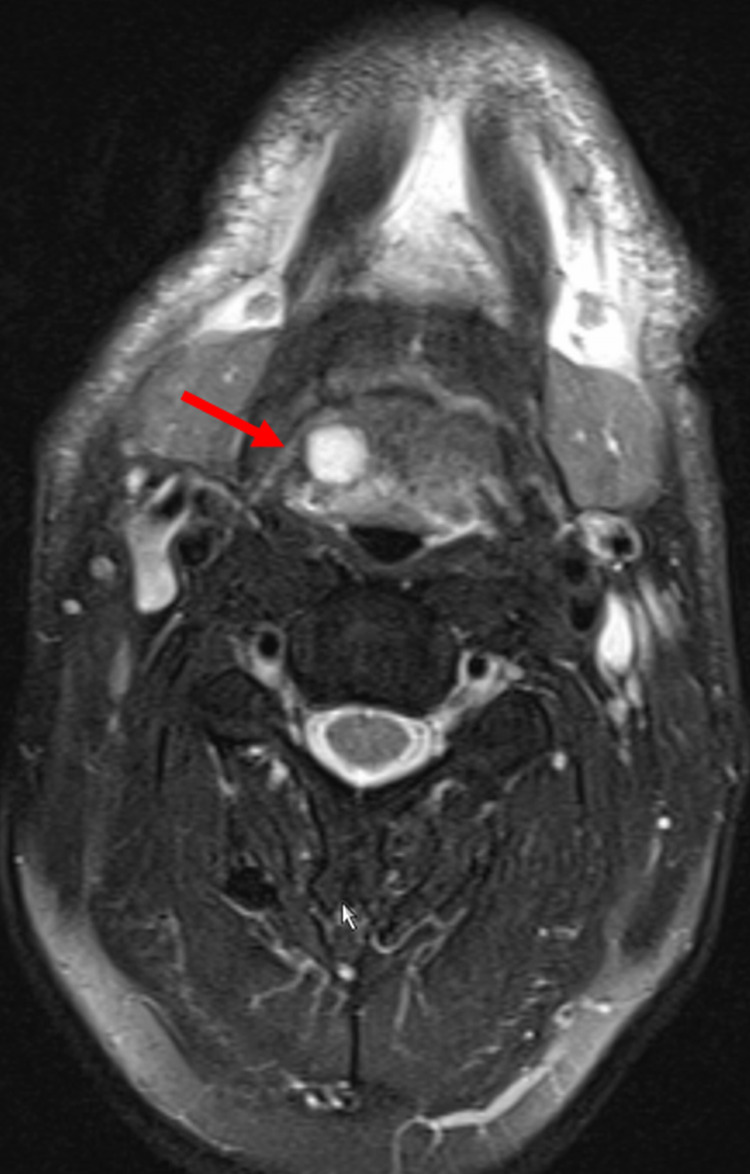
Tongue root cyst in a m.3243A>G carrier Axial, T1-weighted MRI image of the collum showing a hyperintense lesion in the right tongue root, interpreted as thin- and smooth-walled, pre-epiglottic cyst with 1 cm in diameter and protein-rich content, which did not enhance after application of gadolinium.

The patient is a 56-year-old male diagnosed with mitochondrial encephalopathy, lactic acidosis, and stroke-like episodes (MELAS) syndrome due to the variant m.3243A>G in MT-TL1 at the age of 50 years. MELAS manifested phenotypically with recurrent stroke-like episodes, seizures, cognitive decline, ataxia, psychiatric abnormalities, visual impairment, hypoacusis, pre-diabetes, hyperlipidemia, myopathy, and lactic-acidosis. At the age of 56 years, he was admitted for another stroke-like episode. He presented to the emergency department with vertigo, confusion, speech disturbance, difficulties in finding words, impaired concentration, and ataxic gait. Clinical exam revealed weak head anteflexion, cerebellar speech, visual impairment with only light/dark discrimination, hypoacusis, mild ptosis bilaterally, diffuse wasting of the upper limbs, bradydiadochokinesia bilaterally, wasting of the thighs, lower limb ataxia, and reduced Achilles tendon reflexes. There was ataxic stance and gait requiring a walker. Cerebral MRI showed a typical acute, stroke-like lesion (SLL) in an occipito-temporal distribution. In addition to the SLL, cerebral MRI accidentally revealed a cyst on the right side of the tongue root. Revision of imaging studies from a previous hospitalisation revealed that the pre-epiglottic cyst with 1 cm in diameter and protein-rich content had been first detected accidentally on ultrasound and later by MRI already at the age of 50 years (Figure 1). Since then the cyst had remained unchanged regarding morphology and extension. The patient did not report any symptoms attributable to the tongue cyst. He denied speech disturbance or dysphagia. A branchiogenic cyst was excluded due to the atypical location. Zenker’s diverticulum was excluded due to the location and morphology. The previous history was negative for previous trauma, chronic infection, or surgery at the site of the cyst. There was no indication for parasitosis.

## Discussion

The patient is interesting as a tongue cyst has not been described as a manifestation of MELAS before. Anyhow, there are indications that cyst formation is generally more prevalent in patients with a MID than in the general population [2-4], but systematic studies investigating the frequency of cysts in MID patients have not been carried out yet. Cysts in MIDs have been reported in various organs, including the cerebrum, liver, kidneys, pancreas, thyroid gland, ovaries, and bones [2-4]. Recently, subependymal pseudocysts have been reported in a neonate with a MID due to a variant in IBA57 [5]. White matter lesions together with well-delineated cysts have also been reported in a 1-year-old male with a MID due to biallelic variants in ISCA2 [6]. In a patient with maternally inherited diabetes and deafness due to the variant m.3243A>G in MT-TL1 retinal pseudocysts have been detected on optical coherence tomography [7]. Cavitating and tigroid leukoencephalopathy has been reported in a patient carrying an NDUFA2 variant but without systemic biochemical abnormalities of a MID [8]. Multifocal cavitating white matter lesions with a predominant occipital distribution were described in a female with leukodystrophy due to a mitochondrial LYRM7 mutation [9]. In addition to these clinical reports, autopsy studies have shown encephaloclastic lesions, particularly microscopic cysts, in patients with pyruvate-dehydrogenase deficiency and patients with short-chain enoyl-CoA hydratase deficiency [10].

The reason for the presumably increased prevalence of cyst formations in MIDs remains elusive but it can be speculated that it is due to reduced cell adhesion, due to ectopia, or due to abnormal proliferation of cells. It can also be speculated that enhanced focal apoptosis, disturbed mitochondrial fusion/fission, or increased mitophagy was responsible for the focal tissue rarefication. From MIDs, it is well known that the prevalence of benign and malign neoplasms is increased [11]. The reason why the cyst in the index patient developed particularly in the tongue and not in other locations remains speculative. Unfortunately, he did not consent to a biopsy of the cyst to further investigate the etiology of the lesion.

## Conclusions

We present the first case of a MELAS patient with a tongue root cyst, which was suspected to be causally related to the m.3243A>G variant. Though tongue cysts have not been reported as a manifestation of the m.3243A>G variant, there are indications that MIDs carry an increased risk of developing cysts in any organ, including the tongue. Further studies are required to assess if the prevalence of cyst formations is truly increased in MIDs and if cysts of the skeletal or striated muscles also occur in other syndromic or non-syndromic MIDs.
